# Direct Delivery of a Cytotoxic Anticancer Agent into the Metastatic Lymph Node Using Nano/Microbubbles and Ultrasound

**DOI:** 10.1371/journal.pone.0123619

**Published:** 2015-04-21

**Authors:** Takuma Sato, Shiro Mori, Maya Sakamoto, Yoichi Arai, Tetsuya Kodama

**Affiliations:** 1 Graduate School of Biomedical Engineering, Tohoku University, 4–1 Seiryo-machi, Aoba, Sendai, Miyagi, 980–8575, Japan; 2 Department of Oral and Maxillofacial Surgery, Tohoku University Hospital, 1–1 Seiryo-machi, Aoba, Sendai, Miyagi, 980–8575, Japan; 3 Department of Oral Diagnosis, Tohoku University Hospital, 1–1 Seiryo-machi, Aoba, Sendai, Miyagi, 980–8575, Japan; 4 Department of Urology, Tohoku University Graduate School of Medicine, 1–1 Seiryo-machi, Aoba, Sendai, Miyagi, 980–8575, Japan; Istituto Superiore di Sanità, ITALY

## Abstract

Direct injection of an anticancer agent into a metastatic lymph node (LN) has not been used as a standard treatment because evidence concerning the efficacy of local administration of a drug into a metastatic LN has not been established. Here we show that the combination of intralymphatic drug delivery with nano/microbubbles (NMBs) and ultrasound has the potential to improve the chemotherapeutic effect. We delivered *cis*-diamminedichloroplatinum (II) (CDDP) into breast carcinoma cells *in vitro* and found that apoptotic processes were involved in the antitumor action. Next, we investigated the antitumor effect of intralymphatic chemotherapy with NMBs and ultrasound in an experimental model of LN metastasis using MXH10/Mo-*lpr*/*lpr* mice exhibiting lymphadenopathy. The combination of intralymphatic chemotherapy with NMBs and ultrasound has the potential to improve the delivery of CDDP into target LNs without damage to the surrounding normal tissues. The present study indicates that intralymphatic drug delivery with NMBs and ultrasound will potentially be of great benefit in the clinical setting.

## Introduction

Local injection of anticancer agents has not been used as a standard treatment because it causes damage to the surrounding normal tissues. Evidence for the efficacy and safety of local administration of anticancer agents into metastatic lymph nodes (LNs) has not been obtained so far and therefore this approach cannot be regarded as a recommended treatment at the present time. However, when we consider the anatomical characteristics of the lymphatic network, it may be possible to propose a new treatment for LN metastasis [1].

LNs are interposed between the afferent and efferent vessels in the lymphatic network and are surrounded by a capsule of dense connective tissue [2]. Accordingly, in metastatic LNs without extranodal invasion, a capsule of dense connective tissue also surrounds the metastatic cancer cells. Local administration of cytotoxic anticancer agents into a metastatic LN that does not show extranodal invasion should be therapeutically effective, with minimal damage to the surrounding normal tissues [3]. Furthermore, since the velocity of lymph fluid flow in the lymph network is slow compared to blood flow [4], anticancer agents administered into the LN may accumulate in the lymph network for a long time. Therefore, a curative treatment for LN metastasis should be possible using anticancer agents that are administered into the LN, even if the doses of the anticancer agents are small. However, since an appropriate LN metastasis model has not been available until now, it has been difficult to verify the antitumor effect of agents administered directly into metastatic LNs.

Recently, we have established an experimental LN metastasis model using a recombinant inbred strain of mouse, the MXH10/Mo-*lpr/lpr* (MXH10/Mo/lpr) mouse, which shows swelling of LNs that reach sizes similar to those of human LNs [1]. MXH10/Mo/lpr mice exhibit remarkable lymphadenopathy and provide several advantages as an animal model for LN metastasis. These experimental mice permit the study of a tumor confined to a single LN (experimental metastatic LN model), precise and reproducible injection of agents into LNs, and detailed monitoring of any changes in the internal structure of individual LNs [5, 6].

*cis*-Diamminedichloroplatinum (II) (CDDP) has been widely used against many types of solid tumor [7]. The amount of CDDP administered intravenously is limited by dose-related side effects, thus, the concentrations achieved in target LNs may be insufficient for effective antitumor activity. Direct injection of CDDP into the target tissues/organs will produce a higher local concentration than intravenous administration, although extravasation of CDDP into soft tissues may result in local tissue damage. However, LNs are surrounded by a capsule of dense fibrous tissue, and therefore drugs precisely injected into LNs may well remain there without major leakage. The concept is that direct injection of CDDP into the LN will produce a higher delivery dose of drug to the target LN with minimal injury to the surrounding normal tissues.

Ultrasound (US) irradiation in the presence of US contrast agents (i.e., microbubbles) induces transient and reversible membrane poration of target cells that enhances delivery of exogenous molecules and improves the antitumor activity of chemotherapeutic drugs [8]. This approach has significant benefits for cancer therapy, including low toxicity to adjacent normal tissues, high tissue selectivity and repeatable applicability. Furthermore, *in vitro* experiments have demonstrated that US contrast agents combined with US can directly induce apoptosis of tumor cells. Hence, combining US with microbubbles may represent a novel method for enhancing anticancer agent delivery to a desired target site and has the additional advantage of exerting its own antitumor effect [9].

In our preceding report [3], intralymphatic chemotherapy (direct injection of anticancer drugs into LNs) achieved a higher degree of antitumor effect than intravenous chemotherapy against sarcoma cells in LNs. A marked antitumor effect of a combination of intralymphatic chemotherapy and US with nano/microbubbles (NMBs) was also demonstrated.

In the present study, the synergistic antitumor activity of CDDP in the presence of NMBs and US irradiation against breast carcinoma cells was investigated *in vitro*. Moreover, the antitumor efficiency of intralymphatic chemotherapy with NMBs and US was investigated in an experimental metastatic LN model using MXH10/Mo/lpr mice.

## Materials and Methods

All experiments were approved by the Institutional Animal Care and Use Committee of Tohoku University.

### Cell preparation

FM3A (mammary carcinoma) and MH129F (hepatoma) cells were established from C3H/He mice and obtained from the Cell Resource Center for Biomedical Research, Institute of Development, Aging and Cancer, Tohoku University. FM3A-Luc cells, which stably expressed the firefly luciferase gene, were established by electroporation of FM3A cells with pGL4.51 (Invitrogen, Carlsbad, CA, USA) using a Gene Pulser Xcell (Biorad, Hercules, CA, USA). FM3A and MH129F cells were maintained in RPMI 1640 medium (Biological Industries, Kibbutz Beit Haemek, Israel) supplemented with 10% fetal bovine serum (FBS; Thermo Fisher Scientific, Waltham, MA, USA) and 1% penicillin/streptomycin (Sigma-Aldrich, St. Louis, MO, USA). FM3A-Luc cells were cultured in RPMI 1640 medium, supplemented with 10% FBS, 1% penicillin/streptomycin and 1 mg/mL geneticin (G418 sulfate, Sigma-Aldrich). Cells were incubated at 37°C in a mixture of 5% carbon dioxide and 95% air. Before *in vitro* and *in vivo* experiments, the absence of mycoplasma contamination in the cell cultures was ensured using a MycoAlert Mycoplasma Detection Kit (Lonza Rockland, Inc., Rockland, ME, USA), according to the manufacturer's protocol [1].

### Animal model

MXH10/Mo-*lpr*/*lpr* (MHX10/Mo/lpr) mice, established by intercrossing MRL/MpJ-*lpr*/*lpr* (MRL/lpr) and C3H/HeJ-*lpr*/*lpr* strains, were bred and maintained as previously described [1].

### Preparation of NMBs

Acoustic liposomes (ALs), used as NMBs, were composed of 1,2-distearoyl-*sn*-glycero-3-phosphatidylcholine (DSPC) (NOF Co., Tokyo, Japan) and 1,2-distearoyl-*sn*-glycero-3-phosphoethanolamine-methoxy-polyethyleneglycol (DSPE-PEG[2000-OMe]) (NOF Co.) (94:6 mol/mol), containing C_3_F_8_ gas; their preparation has been described in detail previously [10].

### Ultrasound irradiation

A flat, disc-shaped, 1.0-MHz US transducer of 30 mm diameter (BFC Applications, Fujisawa, Japan) was used for irradiation. The US conditions were as previously described [8]. For *in vivo* experiments, the US intensity was 3.0 W/cm^2^, the duty cycle 20% and the exposure time 120 s. For *in vitro* experiments, the US intensity was 0.1, 0.5 or 1.0 W/cm^2^, the duty cycle 50% and the exposure time 10 s. All experiments involving US exposure were carried out in a test chamber filled with tap water heated to 38°C.

### *In vitro* measurement of cell viability

FM3A and MH129F cells were suspended at a concentration of 1.0 × 10^5^ cells/mL in the same medium used for cell preparation and aliquoted (450 μL) into 48-well plates. For the control and US alone groups, 50 μL of phosphate buffered saline (PBS; Sigma-Aldrich) was added; for the US + ALs group, 25 μL of PBS and 25 μL of ALs was added; and for the CDDP + ALs and CDDP + ALs + US groups, 25 μL of CDDP in PBS and 25 μL of ALs was added, with final CDDP concentrations of 0.1, 0.5 or 5.0 μM (FM3A cells) and 0.01, 0.1 or 1.0 μM (MH129F cells). Samples from the US alone, US + ALs and CDDP + ALs + US groups were positioned 100 mm above a 30-mm diameter US transducer and then exposed to US (0.1, 0.5 or 1.0 W/cm^2^). All samples were incubated for 24 h, and the numbers of intact FM3A and MH129F cells were counted by the trypan blue dye exclusion test, using a Countess Automated Cell Counter (Invitrogen). Each experiment was performed using 6 samples. Normalized cell viability was obtained by dividing the value for each treated sample by the mean of the control samples.

### Measurements of the expression levels of apoptosis-related genes

To investigate the possible mechanisms underlying an antitumor action of CDDP + ALs + US against FM3A cells, the expression levels of apoptosis-related genes were quantified in the control, CDDP + ALs, US + ALs and CDDP + ALs + US groups (*n* = 5 for each). The US intensity used was 1.0 W/cm^2^, and the CDDP concentration was 0.5 μM. Samples were treated as described for measurement of cell viability and subsequently incubated for 6 h, and then total RNA extracted from each sample with illustra RNAspin Mini isolation kits (GE Healthcare Japan, Tokyo, Japan), and reverse transcription performed using iScript RT Supermix for RT-qPCR (Biorad), according to the manufacturers’ protocols. For quantitative real-time PCR, all samples were amplified with gene-specific primers using KAPA SYBR FAST qPCR kits (Kapa Biosystems, Woburn, MA, USA), according to the manufacturer’s protocol; reactions were run in duplicate on an MX3000P instrument (Stratagene, Santa Clara, CA, USA). Caspase-3, caspase-8, caspase-9 and p53 gene expression levels were normalized against a reference gene (β-actin) and the fold change determined relative to the control. The PCR primers used were (forward, reverse): murine (m)-β-actin, 5’-GATCATTGCTCCTCCTGAGC-3’, 5’-ACATCTGCTGGAAGGTGGAC-3’; m-caspase-3, 5’-TGAAGGGGTCATTTATGGGACA-3’, 5’-CCAGTCAGACTCCGGCAGTA-3’; m-caspase-8, 5’-CAACTTCCTAGACTGCAACCG-3’, 5’-TCCAACTCGCTCACTTCTTCT-3’; m-caspase-9, 5’-TCCTGGTACATCGAGACCTTG-3’, 5’-AAGTCCCTTTCGCAGAAACAG-3’; m-p53, 5’-GCGTAAACGCTTCGAGATGTT-3’, 5’-TTTTTATGGCGGGAAGTAGACTG-3’.

### Quantification of apoptosis

To analyze quantitatively the apoptosis of FM3A cells induced by the combination of CDDP with US and ALs, annexin V-labeled cells were quantified and considered to be apoptotic cells. Samples were prepared as for the assessment of apoptosis-related genes (*n* = 6 for each group). Briefly, 24 h after treatment, the samples were washed twice with PBS and stained with fluorescein isothiocyanate (FITC)-annexin V (PromoKine, Heidelberg, Germany), according to the manufacturer’s protocol. FITC-annexin V-labeled cells were identified using flow cytometry **(**BD Accuri C6; BD Biosciences, Franklin Lakes, NJ, USA**)**, and the data analyzed using FlowJo software (Tomy Digital Biology, Co., Ltd., Tokyo, Japan).

### Metastatic lymph node model

FM3A-Luc cells, suspended in PBS, were mixed with Matrigel; 40 μL of the cell suspension (final concentration, 1.0 × 10^6^ cells/mL) was injected into the unilateral subiliac LN (SiLN) of each female MXH10/Mo/lpr mouse (age, 16–18 weeks) anesthetized with 2% isoflurane (Abbott Japan Co., Ltd.) in oxygen. The injected SiLN was defined as the tumor-bearing SiLN, and an MXH10/Mo/lpr mouse with an SiLN containing FM3A-Luc cells was considered as the experimental metastatic LN model of breast cancer; the day of inoculation was defined as day 1. Injections were performed under US guidance, using a high-frequency US (HFUS) imaging system (Vevo 770; VisualSonics Inc., Toronto, Ontario, Canada). The number of tumors (tumor-bearing LNs) was 1 and the long axis of the tumor-bearing SiLN was approximately 16 mm (that of the SiLN was approximately 13 mm).

### *In vivo* ultrasound treatment

Sixteen female MXH10/Mo/lpr mice were divided into 4 groups (*n* = 4 for each): control, US + ALs, CDDP + ALs and CDDP + ALs + US. 20 μL of ALs mixed with either 40 μL of saline (control and US + ALs groups) or 0.75 μg/g bodyweight of CDDP in 40 μL of saline (CDDP + ALs and CDDP + ALs + US groups) was injected directly into the tumor-bearing SiLN on days 3, 4 and 7. Drugs (including ALs) were injected into the tumor-bearing SiLN using a 30-gauge insulin syringe with a permanently attached needle under US guidance (HFUS imaging system), and confirmed to spread diffusely throughout the LN. In the US + ALs and CDDP + ALs + US groups, the tumor-bearing SiLN was positioned 100 mm above a 30-mm diameter US transducer and exposed to US 30 s after administration of the agents (exposure time, 120 s). Each mouse was anesthetized with 2.0% isoflurane in oxygen. During the experiments, monitoring of animal health (measurement of body weight and the size of the tumor-bearing SiLN, eating behavior, drinking behavior, body waste, and the condition of the skin and hair coat) was carried out every day. Analgesics (non-steroidal anti-inflammatory drugs) were available for administration to any mice that experienced pain, although none were actually needed. All animal experiments were terminated and a euthanasia procedure (cervical dislocation under general anesthesia) was carried out when any of the following abnormalities were found: a marked increment in the size of the tumor-bearing SiLN (a 10% increase in body weight); body weight loss (20% or more); food and water intake difficulties; diarrhea or bleeding over prolonged periods; rough hair coat; progressive dermatitis; abnormal posture; self-induced trauma; or respiratory distress. However, no mice in the present study exhibited any of the aforementioned abnormalities during the experimental period and were euthanized before the last day of the experiments (day 10).

### Monitoring of tumors in the SiLN by measurement of luciferase activity

*In vivo* bioluminescence imaging was performed using an IVIS Lumina system (Xenogen Co., Alameda, CA, USA). Each mouse was anesthetized with 2.0% isoflurane in oxygen. On days 3, 5, 8 and 10, luciferase activity was quantified as previously described [3] and (for each group) normalized to that on day 3.

### Quantification of blood vessel densities in the tumor-bearing SiLNs using contrast-enhanced HFUS imaging

Tumor-bearing SiLNs were visualized using a contrast-enhanced HFUS (CE-HFUS) imaging system with a 25 MHz center-frequency transducer (RMV-710B), on day 0 (the day before inoculation), day 6 and day 9. Sonazoid (Daiichi Sankyo, Tokyo, Japan), used as the microbubbles for imaging, was prepared according to the manufacturer’s instructions. Two-dimensional consecutive images of the tumor-bearing SiLN (slice thickness, 100 μm) were collected before and after injection of 100 μL Sonazoid into the tail vein. The differences in the video intensities between pre-injection and post-injection image frames were measured and areas exceeding a set threshold value were considered to be vessel extraction images. The images were reconstructed three-dimensionally to obtain the blood vessel density of each tumor-bearing SiLN as previously described [5]. The vessel densities of the tumor-bearing SiLNs were normalized to values obtained on day 0.

### Histological analysis

On day 10, cervical dislocation euthanasia was carried out under inhalation anesthesia and subsequently samples were resected, fixed, dehydrated, embedded in paraffin and cut into 4-μm serial sections. Sections were stained with hematoxylin and eosin (HE), and immunostained with anti-CD31 antibody to detect CD31-positive cells as described previously [3]. To obtain the blood vessel density in CD31-immunostained sections from the 4 groups, the densities of the macrovessels (defined as CD31-stained vascular structures with a diameter ≥30 μm) and microvessels (CD31-stained vascular structures with a diameter ranging from 5 to 30 μm) were measured as previously described [3]. To determine the density of the macrovessels, CD31-stained vascular structures were manually traced, then their dimensions were measured and totalized. The summed value was divided by the total area of the specimen using commercially available software (Photoshop C4 Extended; Adobe Systems, Inc., San Jose, CA, USA). The specimen boundary was measured with the aid of a low magnification (× 40) microscope (BX43; Olympus Co., Tokyo, Japan). The microvessel density was measured using the ‘hot-spot’ method [11]. Four hot-spot fields with the highest microvessel density were analyzed under low magnification (× 40), and then the dimensions of the CD31-immunostained vascular structures were measured under high magnification (× 200) using the same method as described for macrovessel density (see above). The microvessel density was calculated by dividing the total microvessel area, consisting of vasculature with a minor axis of 5–30 μm, by the total field size of the CD31-positive hot-spot area (416 μm × 312 μm), using Photoshop C4 Extended software.

### Statistical analysis

Values are expressed as the mean ± SEM (standard error of the mean). Comparisons between groups were made using analysis of variance (ANOVA) and the Tukey-Kramer test. Differences were considered to be significant at *P* < 0.05. Statistical analysis was performed using JMP 10 software (SAS Institute Inc., Cary, NC, USA).

## Results

### *In vitro* ultrasound treatment

**Fig 1:**
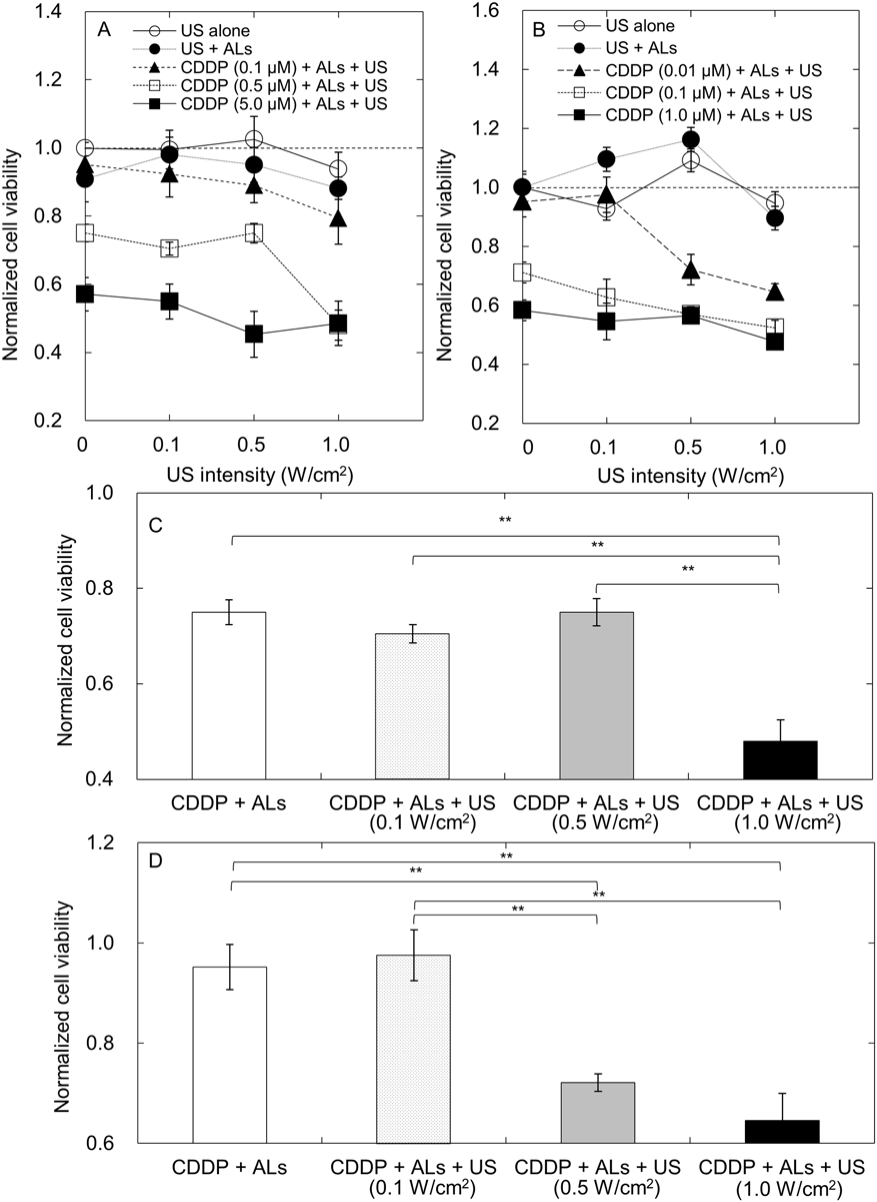
*In vitro* cell viability. Normalized cell viability was obtained by dividing the value in each group by the mean value of the control samples (*n* = 6 for each group). (A) Normalized cell viability of FM3A cells. (B) Normalized cell viability of MH129F cells. (C) Normalized cell viability of FM3A cells when the level of CDDP was 0.5 μM. A significant difference in normalized cell viability between the CDDP + ALs and CDDP + ALs + US groups was demonstrated only at an US intensity of 1.0 W/cm^2^. (D) Normalized cell viability of MH129F cells when the concentration of CDDP was 0.01 μM. There was a significant difference in normalized cell viability between the CDDP + ALs and CDDP + ALs + US groups at US intensities of 0.5 and 1.0 W/cm^2^. Mean ± SEM values are shown. **P < 0.01.

Cell viability depends on the concentration of CDDP used and the US intensity applied in the presence of microbubbles [9]. First, we investigated the relationship between normalized cell viability and US intensity (Fig 1A and 1B). FM3A and MH129F cell viability decreased with increasing CDDP concentration, but varied little with US intensity in the range 0.1 to 1.0 W/cm^2^, except at a CDDP concentration of 0.5 μM (FM3A cells) and 0.01 μM (MH129F cells). Fig 1C shows the normalized FM3A cell viability at a CDDP concentration of 0.5 μM. The normalized cell viability was significantly lower in the CDDP + ALs + US group than in the CDDP + ALs group, only when the US intensity was 1.0 W/cm^2^ and the CDDP concentration was 0.5 μM. MH129F cell viability was significantly lower in the CDDP + ALs + US group than in the CDDP + ALs group when the US intensity was either 0.5 or 1.0 W/cm^2^ and the CDDP concentration was 0.01 μM (Fig 1B and 1D).

### Measurements of the expression levels of apoptosis-related genes

**Fig 2:**
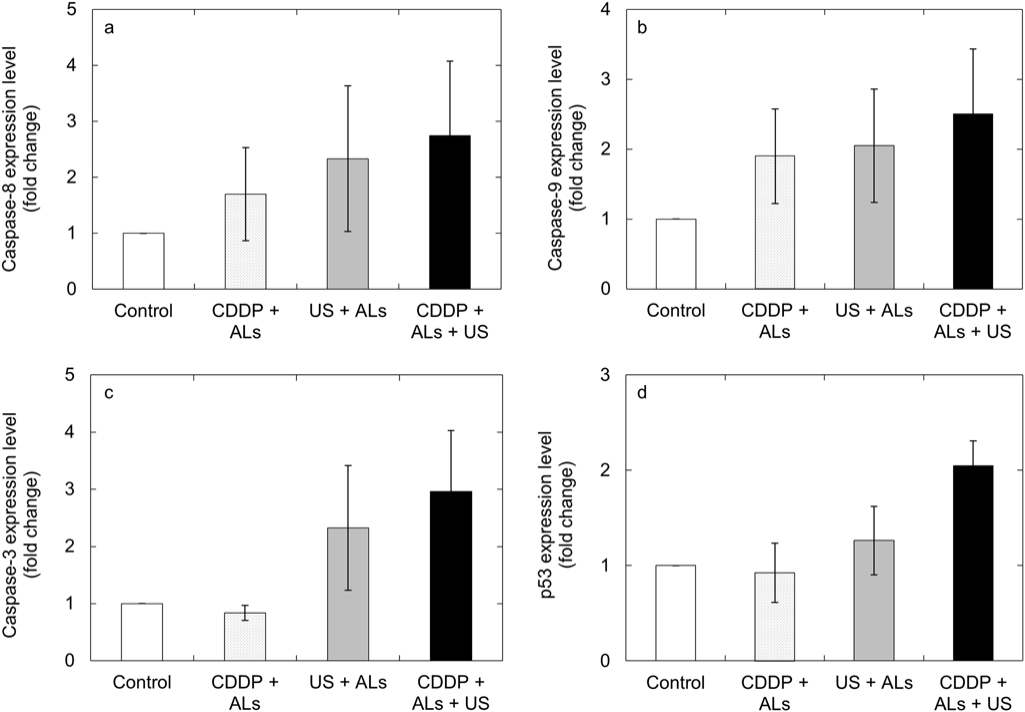
The expression levels of apoptosis-related genes. The mean fold-change in the expression level of each gene, relative to the control group, was measured for the US + ALs, CDDP + ALs and CDDP + US + ALs groups (*n* = 5 for each group). Expression levels of caspase-8 (a), caspase-9 (b), caspase-3 (c) and p53 (d) in FM3A cells. There was a trend towards higher expression of all 4 genes in the CDDP + ALs + US group compared with the other 3 groups. Mean ± SEM values are shown.

Next, we investigated whether the expression levels of 4 apoptosis-related genes were increased by CDDP + ALs + US. There was a trend towards higher expression levels of caspase-3, caspase-8, caspase-9 and p53 in the CDDP + ALs + US group (US intensity, 1.0 W/cm^2^; CDDP concentration, 0.5 μM), compared with the other 3 groups (Fig 2a, 2b, 2c and 2d); however, statistically significant differences were not demonstrated.

### Quantification of apoptosis

**Fig 3:**
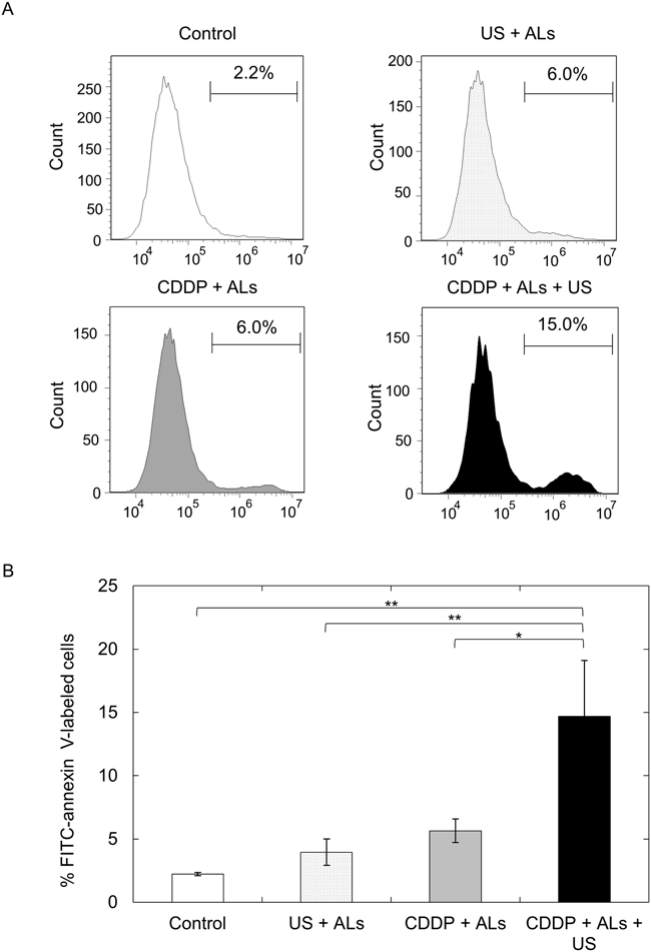
Quantification of apoptosis. (A) Flow cytometric analysis of FITC-annexin V-labeled FM3A cells. The percentage of FM3A cells labeled with FITC-annexin V is shown. The abscissa indicates the FITC fluorescence intensity (arbitrary units). (B) The percentage of FITC-annexin V-labeled FM3A cells in each experimental group (*n* = 6). The number of FITC-annexin V-labeled cells in the CDDP + ALs + US group was significantly higher than that in the control (*P* < 0.01), US + ALs (*P* < 0.01) and CDDP + ALs (*P* < 0.05) groups. Mean ± SEM values are shown. *P < 0.05; **P < 0.01.

The proportion of FITC-annexin V-labeled cells, determined from flow cytometric analysis, was significantly higher in the CDDP + ALs + US group than in the control (*P* < 0.01), US + ALs (*P* < 0.01) and CDDP + ALs (*P* < 0.05) groups (Fig 3A and 3B).

### Comparison of treatment efficacy by measurement of luciferase activity

**Fig 4:**
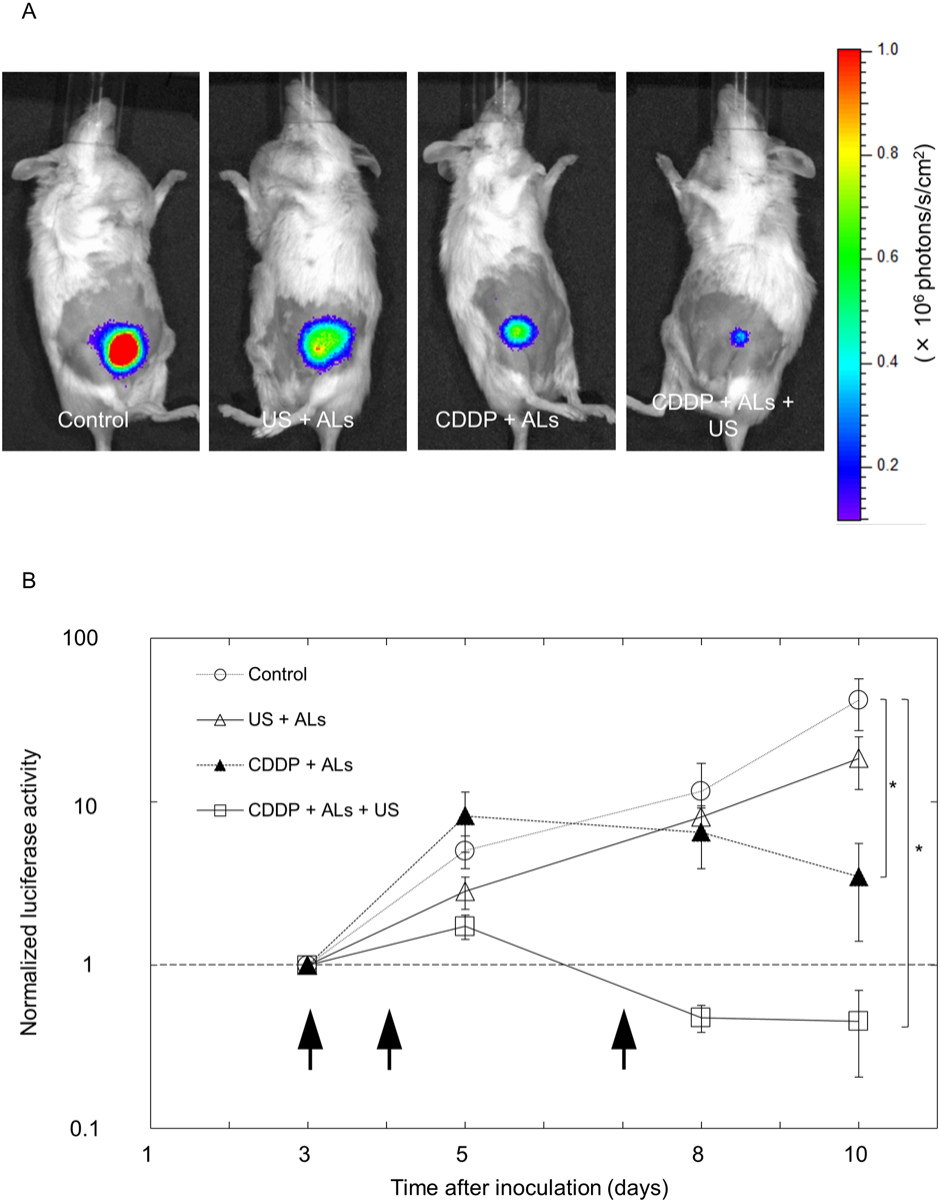
Evaluation of the treatment efficacy of delivery of CDDP with ALs and US. (A) *In vivo* bioluminescence imaging on day 10. (B) Longitudinal analysis of normalized luciferase activity (*n* = 4 for each group). Values at different time points were normalized to those on day 3. Black arrows indicate the days on which treatment was administered (days 3, 4 and 7). On day 10, normalized luciferase activity in the CDDP + ALs + US group was significantly lower than that in the control group (*P* < 0.05) and showed a trend towards being lower than that in the US + ALs or CDDP + ALs group. Mean ± SEM values are shown. *P < 0.05.

The *in vitro* experiments for FM3A cells described above demonstrated that under particular conditions, US and ALs enhanced the antitumor effect of CDDP, which involved an apoptotic process. Next, we evaluated the antitumor activity exerted by combining intralymphatic CDDP injection with US and ALs *in vivo*, using MXH10/Mo/lpr mice with tumor-bearing SiLNs. Fig 4A shows representative bioluminescence images obtained on day 10. The normalized luciferase activity in the CDDP + ALs + US group was significantly lower than that in the control group on day 10 (Fig 4B). There was also a trend towards a lower luciferase activity in the CDDP + ALs + US group compared to the US + ALs and CDDP + ALs groups, although statistical significance was not reached.

### Quantification of blood vessel density in tumor-bearing SiLNs

**Fig 5:**
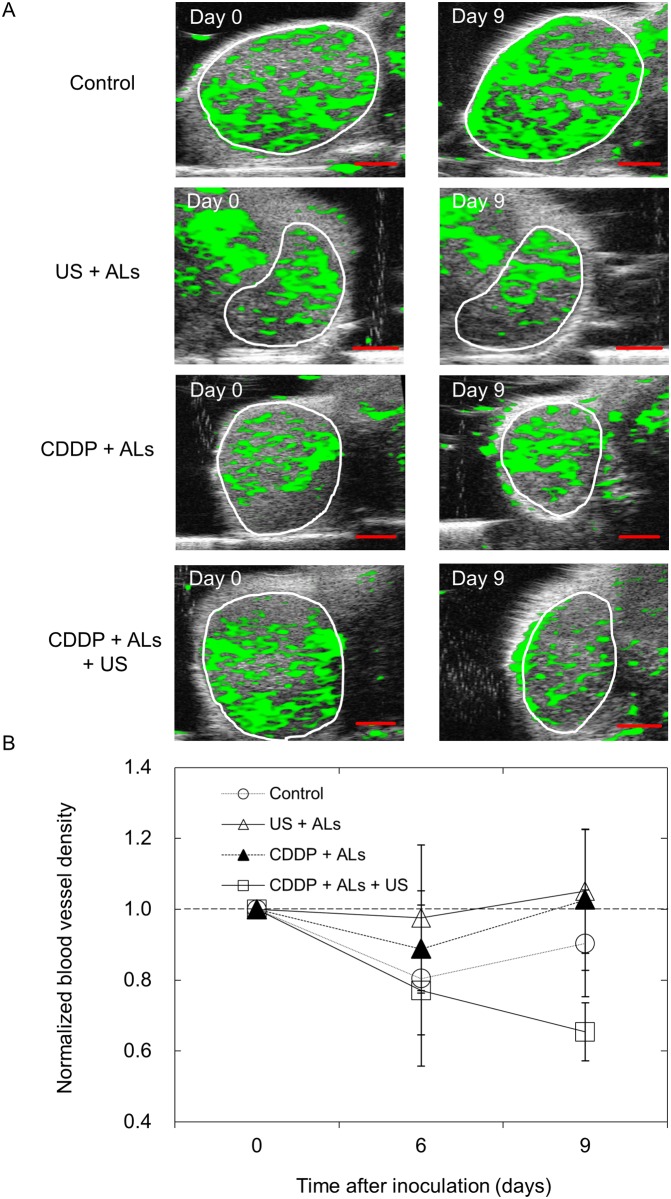
Blood vessel extraction images of tumor-bearing SiLNs. (A) Representative vascular extraction images for each group, taken from the widest cross-section of the tumor-bearing SiLN. The white circles indicate the boundaries of each SiLN. In the control, US + ALs and CDDP + ALs groups, no obvious changes in vessel structures were observed between days 0 and 9. In the CDDP + ALs + US group, the proportion of the tumor-bearing SiLN colored green in the vascular extraction image was lower on day 9 than on day 0. (B) Normalized blood vessel densities of tumor-bearing SiLNs (*n* = 4 for each group). Values at different time points were normalized to those on day 0. There was a trend for normalized blood vessel density on day 9 to be lower in the CDDP + ALs + US group than in the other 3 groups, although there were no significant differences between any of the groups. Mean ± SEM values are shown. Scale bars represent 2 mm.

To investigate the relationship between changes in blood vessel density and either tumor growth or therapeutic intervention, a longitudinal analysis of blood vessel densities in the tumor-bearing SiLNs was carried out using 3D CE-HFUS imaging (Fig 5A). The normalized blood vessel density of the CDDP + ALs + US group progressively decreased, whereas only minor changes were observed between day 0 and day 9 in the other groups (Fig 5B).

### Histopathology analysis

**Fig 6:**
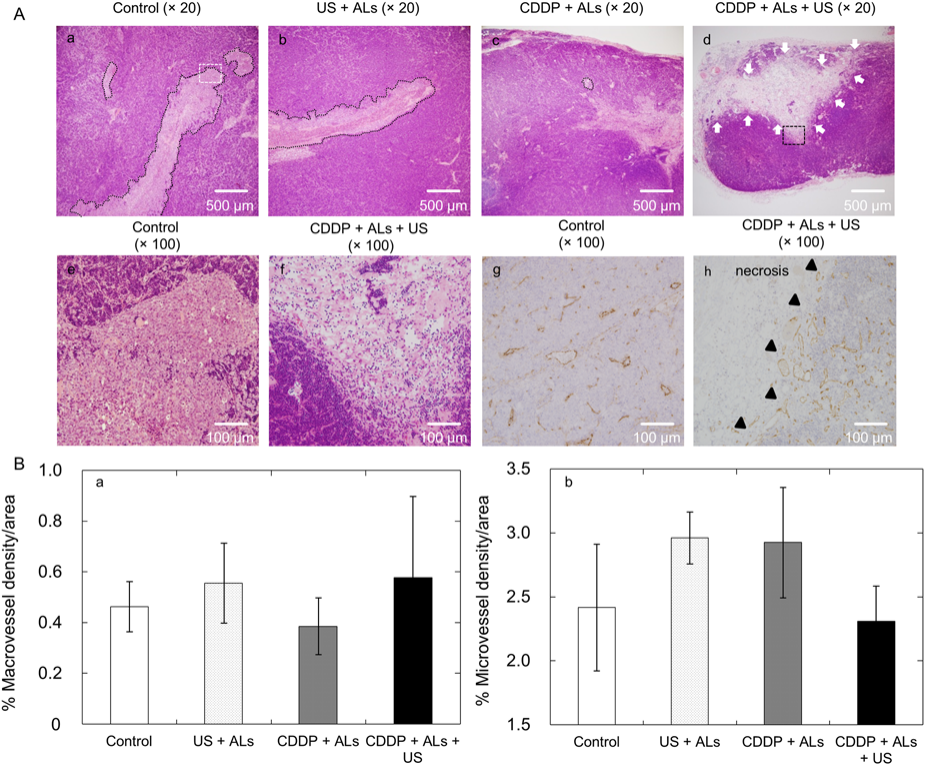
Histopathology analysis. (A) Representative sections stained with HE are shown for (Aa) control, (Ab) US + ALs, (Ac) CDDP + ALs, and (Ad) CDDP + ALs + US groups. Panels (Ae) and (Af) show higher magnification views of the regions highlighted in panels (Aa) (white dotted square) and (Ad) (black dotted square), respectively. CD31-immunostained sections are shown for the control (Ag) and CDDP + ALs + US (Ah) groups. Black dotted lines indicate carcinomatous lesions and white arrows indicate necrotic lesions. Black triangles indicate the border between normal tissue and necrotic areas. In the control and US + ALs groups, widespread cancerous lesions can be seen (Aa, b), while smaller lesions were evident in sections from the CDDP + ALs group (Ac). In sections from the CDDP + ALs + US group, carcinomatous lesions could not be detected, but necrotic lesions were observed (Ad, f). In the control group, CD31-positive vasculature was distributed over the entire lesion (Ag), whereas in the CDDP + ALs + US group CD31-positive vasculature was rarely observed in necrotic lesions (Ah). (B) Quantification of macrovessel and microvessel densities of tumor-bearing SiLNs. No significant differences in macrovessel and microvessel densities were detected between any of the experimental groups.

Tumor cells aligned in a longitudinal direction were observed in HE-stained sections taken from the control and US + ALs groups (the major axis of the tumor in both groups was approximately 3 mm), while only small tumors were found in the CDDP + ALs group (Fig 6Aa, 6Ab and 6Ac). The reason why tumor cells in the control and US + ALs groups expressed the aforementioned unique growth pattern remains unknown. In the CDDP + ALs + US group, tumor cells were not obviously present and circumscribed necrotic lesions were observed in the HE-stained sections (Fig 6Ad and 6Af). CD31-immunostained sections from the CDDP + ALs + US group revealed a decrease in CD31-positive vascular structures within the necrotic lesions (Fig 6Ah). The densities of the macrovessels and microvessels in the CD31-immunostained sections obtained from each group were analyzed. Hot-spots (areas rich in vascular-structures) were detected only in normal lymphatic tissues (including peritumoral areas), and not in intratumoral areas. Fig 6B shows that there were no significant differences in the densities of macrovessels and microvessels between the four groups.

## Discussion

Adequate access to target cells/tissues is important to enable a chemotherapeutic drug to exert a meaningful antitumor action [12]. The intravenous route is most commonly used for anticancer drug administration, but this is associated with drug distribution throughout the entire body and difficulties in accessing target sites, especially the lymphatic system [13, 14]. The physiological features of the lymphatic system permit drugs injected intralymphatically to be retained at high concentrations for prolonged periods; indeed, intralymphatic chemotherapy is superior to intravenous chemotherapy for the treatment of tumors in LNs [3]. Direct injection of an antitumor drug is a method for improving drug delivery to the target site, but extravasation of many types of drug, including CDDP, can induce severe soft tissue injury [15]. In the present study, obvious loss of body weight was not detected in the mice of any group and grossly visible damage was not observed in the treated LNs and surrounding tissues. Additionally, histopathological findings showed that combining intralymphatic administration of CDDP with US and NMBs produced a prominent and target-specific cytotoxic effect in the target LN without obvious damage to the surrounding normal tissues. The safety and efficacy of direct injections of chemotherapeutic agents into enlarged cervical metastatic LNs have been reported previously [16]. When the desired target LNs are visualized by US imaging techniques, US-guided intralymphatic injection allows the accurate injection of antitumor drugs into them. Additionally, US irradiation in the presence of NMBs may well enhance the antitumor activity of intralymphatic chemotherapy. The combination of these techniques has the potential to overcome some of the limitations of intravenous chemotherapy for the treatment of metastatic LNs in patients with head and neck cancer, melanoma and breast cancer.

US and ALs enhanced the antitumor activity of CDDP but only for particular combinations of US intensities and CDDP concentrations (Fig 1). The main mode of action of CDDP is the induction of apoptosis in a dose- and time-dependent manner [17, 18]. Apoptosis is also induced by US and microbubbles [19] in an US intensity-dependent manner [20]. US irradiation with NMBs or injection of CDDP alone failed to induce apoptosis of FM3A cells, whereas the combination of both procedures successfully induced apoptosis. Thus, although US at an intensity of 1.0 W/cm^2^ and CDDP at a concentration of 0.5 μM were not sufficient alone to induce apoptosis of FM3A cells, they acted synergistically to trigger apoptosis. However, the percentage of annexin V-labeled FM3A cells (considered to be apoptotic cells) in the CDDP + ALs + US group (CDDP concentration, 0.5 μM; US intensity, 1.0 W/cm^2^) was approximately 15% (Fig 3B), suggesting that other mechanisms might exert an influence on the decrease in cell viability (approximately 50%) observed in our viability experiments for FM3A cells (Fig 1A and 1C) [21]. Caspase-8 and caspase-9 are essential proteases in the extrinsic and intrinsic apoptotic pathways, respectively [22], but major changes in their expression levels were not demonstrated (Fig 2a and 2b). In the present study, measurement of changes in the expression levels of 4 apoptosis-related genes over time and the assessment of factors concerning one trigger of apoptosis (e.g. DNA double strand breaks) were not performed. Further experiments carrying out time-course analysis of apoptosis-related factors will be undertaken in the future.

Although marked antitumor activity against tumors in LNs was observed in the CDDP + ALs + US group, as verified by histopathological analysis, there was no significant difference in normalized luciferase activity between the CDDP + ALs and CDDP + ALs + US groups (Figs 4 and 6). This result indicates that the enhancement of the antitumor action of CDDP by US with NMBs seen in the *in vitro* cell viability experiments was not observed in the *in vivo* experiments. The US intensity and frequency were optimized in our previous study [8]. In order to obtain maximum antitumor activity on a LN tumor with a minimal dose of CDDP, the optimal injection dose of CDDP needs to be determined. US imaging is useful in the diagnosis of metastatic LNs [23], but early diagnosis of LN metastasis by imaging alone is thought to be challenging [5]. A previous study, using CE-HFUS imaging to perform a longitudinal analysis of the blood vessel densities of LNs, found that the growth of a luciferase-labeled tumor in a LN could be observed before the size of the tumor-containing LN had increased [5]. In the present study, a progressive decrease in the blood vessel density of the tumor-bearing SiLN was detected only in the CDDP + ALs + US group (Fig 5B).

Fig 6B shows that US, CDDP and their combination did not affect the structural density of vasculature in the normal lymphatic tissues and that, although not clearly quantified, a decrease in CD31-positive vascular structures in the necrotic area was found in the CDDP + ALs + US group. These results suggest that necrosis may be one major cause of the decrease in blood vessel density measured by CE-HFUS imaging. However, in images of tumor-bearing SiLNs, a circumscribed decrease in green overlay (considered to represent blood flow) was not detected in the areas corresponding to the necrotic lesions shown in HE-stained sections from the CDDP + ALs + US group, and a relatively homogenous decrease was observed. These results suggest that CDDP and US with NMBs may cause a decrease in overall blood flow, but this was not investigated further in the present study.

Several limitations of our present research should be noted. Although measurement of luciferase activity is well suited to the longitudinal analysis of tumor burden in a quantitative manner [24], a correlation between LN luciferase activity and tumor load was not clearly demonstrated. In addition, although it is possible that direct injection of tumor cells into LNs can lead to enhanced immunogenicity of these cells [25, 26], our histopathological analysis showed that tumor growth occurred in the control group whereas necrosis occurred in the CDDP + ALs + US group. Therefore, it is clear that the significant difference in normalized luciferase activity between the control and CDDP + ALs + US groups was not attributable to the immunogenic potential of the tumor cells.

In conclusion, a combination of intralymphatic chemotherapy with US and NMBs has the potential to improve drug delivery to target LNs and achieve a superior chemotherapeutic effect in an experimental metastatic LN model of breast carcinoma, without causing obvious damage to the surrounding normal tissues.

## Supporting Information

S1 Dataset Dataset of Figures. (XLSX)

## References

[1] Shao L, Mori S, Yagishita Y, Okuno T, Hatakeyama Y, Sato T, et al Lymphatic mapping of mice with systemic lymphoproliferative disorder: usefulness as an inter-lymph node metastasis model of cancer. J Immunol Methods. 2013;389(1–2):69–78. 10.1016/j.jim.2013.01.004 23328410

[2] Ross MH, Pawlina W. Histology: a text and altas. 5th ed Philadelphia: Lippincott Williams & Wilkins; 2003.

[3] Sato T, Mori S, Arai Y, Kodama T. The combination of intralymphatic chemotherapy with ultrasound and nano-/microbubbles is efficient in the treatment of experimental tumors in mouse lymph nodes. Ultrasound Med Biol. 2014;40(6):1237–49. 10.1016/j.ultrasmedbio.2013.12.012 24656719

[4] Margaris KN, Black RA. Modelling the lymphatic system: challenges and opportunities. J R Soc Interface. 2012;9(69):601–12. 10.1098/rsif.2011.0751 22237677 PMC3284143

[5] Li L, Mori S, Kodama M, Sakamoto M, Takahashi S, Kodama T. Enhanced sonographic imaging to diagnose lymph node metastasis: importance of blood vessel volume and density. Cancer Res. 2013;73(7):2082–92. 10.1158/0008-5472.CAN-12-4200 23333937

[6] Li L, Mori S, Sakamoto M, Takahashi S, Kodama T. Mouse model of lymph node metastasis via afferent lymphatic vessels for development of imaging modalities. PLoS One. 2013;8(2):e55797 10.1371/journal.pone.0055797 23405215 PMC3565997

[7] Timmer-Bosscha H, Mulder NH, de Vries EG. Modulation of cis-diamminedichloroplatinum(II) resistance: a review. Br J Cancer. 1992;66(2):227–38. 1503895 10.1038/bjc.1992.249 PMC1977827

[8] Kodama T, Aoi A, Watanabe Y, Horie S, Kodama M, Li L, et al Evaluation of transfection efficiency in skeletal muscle using nano/microbubbles and ultrasound. Ultrasound Med Biol. 2010;36(7):1196–205. 10.1016/j.ultrasmedbio.2010.04.016 20620706

[9] Watanabe Y, Aoi A, Horie S, Tomita N, Mori S, Morikawa H, et al Low-intensity ultrasound and microbubbles enhance the antitumor effect of cisplatin. Cancer Sci. 2008;99(12):2525–31. 10.1111/j.1349-7006.2008.00989.x 19018767 PMC11159926

[10] Kodama T, Tomita N, Horie S, Sax N, Iwasaki H, Suzuki R, et al Morphological study of acoustic liposomes using transmission electron microscopy. J Electron Microsc (Tokyo). 2010;59(3):187–96. 10.1093/jmicro/dfp056 19906662

[11] de Jong JS, van Diest PJ, Baak JP. Hot spot microvessel density and the mitotic activity index are strong additional prognostic indicators in invasive breast cancer. Histopathology. 2000;36(4):306–12. 10759944 10.1046/j.1365-2559.2000.00850.x

[12] Minchinton AI, Tannock IF. Drug penetration in solid tumours. Nat Rev Cancer. 2006;6(8):583–92. 16862189 10.1038/nrc1893

[13] Allen TM, Cullis PR. Drug delivery systems: entering the mainstream. Science. 2004;303(5665):1818–22. 15031496 10.1126/science.1095833

[14] Xie Y, Bagby TR, Cohen MS, Forrest ML. Drug delivery to the lymphatic system: importance in future cancer diagnosis and therapies. Expert Opin Drug Deliv. 2009;6(8):785–92. 10.1517/17425240903085128 19563270 PMC3102644

[15] Dorr RT. Antidotes to vesicant chemotherapy extravasations. Blood Rev. 1990;4(1):41–60. 2182147 10.1016/0268-960x(90)90015-k

[16] Osaki T, Ueta E, Yoneda K, Yamamoto T. Intranodal injection of anticancer drugs into fixed cervical metastatic lymph nodes. Oral Dis. 1997;3(4):247–53. 9643221 10.1111/j.1601-0825.1997.tb00050.x

[17] Okamura M, Hashimoto K, Shimada J, Sakagami H. Apoptosis-inducing activity of cisplatin (CDDP) against human hepatoma and oral squamous cell carcinoma cell lines. Anticancer Res. 2004;24(2B):655–61. 15161008

[18] Wang X, Martindale JL, Holbrook NJ. Requirement for ERK activation in cisplatin-induced apoptosis. J Biol Chem. 2000;275(50):39435–43. 10993883 10.1074/jbc.M004583200

[19] Zhong W, Sit WH, Wan JM, Yu AC. Sonoporation induces apoptosis and cell cycle arrest in human promyelocytic leukemia cells. Ultrasound Med Biol. 2011;37(12):2149–59. 10.1016/j.ultrasmedbio.2011.09.012 22033133

[20] Furusawa Y, Zhao QL, Hassan MA, Tabuchi Y, Takasaki I, Wada S, et al Ultrasound-induced apoptosis in the presence of Sonazoid and associated alterations in gene expression levels: a possible therapeutic application. Cancer Lett. 2010;288(1):107–15. 10.1016/j.canlet.2009.06.029 19646810

[21] Gonzalez VM, Fuertes MA, Alonso C, Perez JM. Is cisplatin-induced cell death always produced by apoptosis? Mol Pharmacol. 2001;59(4):657–63. 11259608 10.1124/mol.59.4.657

[22] Elmore S. Apoptosis: a review of programmed cell death. Toxicol Pathol. 2007;35(4):495–516. 17562483 10.1080/01926230701320337 PMC2117903

[23] Toriyabe Y, Nishimura T, Kita S, Saito Y, Miyokawa N. Differentiation between benign and metastatic cervical lymph nodes with ultrasound. Clin Radiol. 1997;52(12):927–32. 9413967 10.1016/s0009-9260(97)80226-7

[24] Klerk CP, Overmeer RM, Niers TM, Versteeg HH, Richel DJ, Buckle T, et al Validity of bioluminescence measurements for noninvasive in vivo imaging of tumor load in small animals. Biotechniques. 2007;43(1 Suppl):7–13, 30 17936938 10.2144/000112515

[25] Martinez-Gomez JM, Johansen P, Erdmann I, Senti G, Crameri R, Kundig TM. Intralymphatic injections as a new administration route for allergen-specific immunotherapy. Int Arch Allergy Immunol. 2009;150(1):59–65. 10.1159/000210381 19339803

[26] Johansen P, Haffner AC, Koch F, Zepter K, Erdmann I, Maloy K, et al Direct intralymphatic injection of peptide vaccines enhances immunogenicity. Eur J Immunol. 2005;35(2):568–74. 15682446 10.1002/eji.200425599

